# Identification and Characterization of MicroRNAs in *Macaca fascicularis* by EST Analysis

**DOI:** 10.1155/2012/957607

**Published:** 2012-07-05

**Authors:** Hao Yang, Haiyang Zhang, Lin Zhu, Chenyu Zhang, Donghai Li

**Affiliations:** ^1^Jiangsu Engineering Research Center for MicroRNA Biology and Biotechnology, State Key Laboratory of Pharmaceutical Biotechnology, School of Life Sciences, Nanjing University, Nanjing 210093, China; ^2^Institute of Discovery Biology, Jiangsu Simcere Pharmaceutical R&D Co., Ltd., 699-18 Xuan Wu Avenue, Nanjing 210042, China

## Abstract

MicroRNAs (miRNAs) are small noncoding RNAs which repress gene expression at the posttranscriptional level. In this study, an expressed sequence tag (EST)-based combined method was applied for the detection of miRNAs in *Macaca fascicularis* which is used as a model animal extensively in medical experiments, particularly those involved with neuroscience and disease. Initially, previously known miRNA sequences from metazoans were used to blast with the EST databases of *Macaca fascicularis*, and then a range of filtering criteria was conducted to remove some pseudo ones. At last a total of 8 novel conserved miRNAs were identified; their functions were further predicted and analyzed. Together, our study provides insight into miRNAs and their functions in *Macaca fascicularis*, indicating that the EST analysis is an efficient and affordable alternative approach for identifying novel miRNA candidates.

## 1. Introduction 

A new class of RNA regulatory genes known as microRNAs (miRNAs) has been found to introduce a whole new layer of gene regulation in eukaryotes [1]. miRNAs are a family of approximate 16~25 nt non-coding single-stranded RNAs that act as post-transcriptional regulators, which have been reported to be located mostly within non-coding regions of genomes in animals, plants, and fungi, and usually transcribed by RNA polymerase II [2–6]. The generation of mature miRNA is a complicated enzyme-participated process, from the initial transcript of pri-miRNA to pre-miRNA with a characteristic hairpin structure [7], then a double-strand miRNA:miRNA*, followed by the assemblage of miRISC complex [3]. miRNA genes constitute a significant group of post-transcriptional regulators which are involved in several physiological or pathological cellular processes.

*Macaca fascicularis (M. fascicularis)*, long tailed and crab eating, is found in Southeast Asia from Burma to the Philippines and southward through China, India, Malaysia, and Indonesia. It is a kind of crucial model animal, which has been used extensively in medical experiments such as the disease model of diabetes [8], as well as those connected with neuroscience [9, 10], because it shares a very similar physiological process with humans. It has also been identified as a possible ideal experiment animal for the vaccine of Ebola virus [11] and monkeypox [12–14] and a known carrier of monkey B virus (cercopithecine herpesvirus) [15].

Although it is deficient in the genome information of *M. fascicularis*, published EST databases in GenBank have made it available to obtain more genetic information. In this study, we used the characteristic features of previously known metazoan miRNAs to systematically search the conserved *M. fascicularis* miRNA homologues in the publicly available EST database. We also predicted and analyzed miRNA-regulated genes in *M*. *fascicularis*. A total of 8 conserved miRNAs were identified, and many miRNA-targeted genes were also predicted. Novel miRNAs were discovered for the purpose of understanding their function in regulating growth, development, metabolism, and other physiological or pathological processes of *M. fascicularis.*

## 2. Materials and Methods

### 2.1. Sequences and Software

We firstly downloaded the known animal miRNA sequences from various species of metazoan from the miRNA database miRBase (Release 14 Sept 2010). After removing the repeated items, the sequences left were considered as the reference set. At last, the total 164,127 EST sequences of *M*. *fascicularis* were downloaded from GenBank; Blast-2.2.24 was downloaded from NCBI and set up locally. What is more, the secondary structure of the pre-miRNAs and the minimal folding free energy were calculated by a web server called mfold [16], followed by the application of software BioEdit with the purpose of improving the analysis efficiency.

### 2.2. Prediction of * M. fascicularis* miRNAs

**Figure 1:**
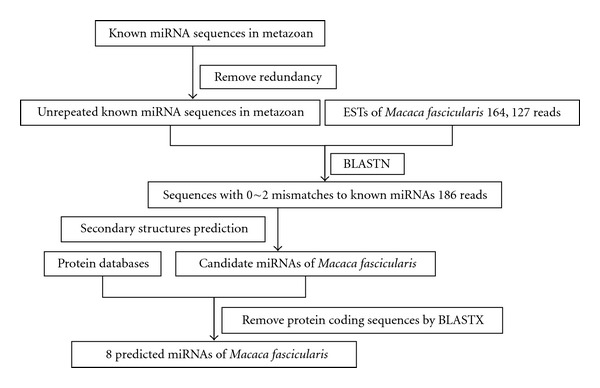
Flowchart of *M*. *fascicularis miRNAs prediction. *

The prediction process was shown in Figure 1. We set the sequences of metazoan miRNAs as queries for BLAST searching against the *M. fascicularis* EST databases, with the BLAST parameters E-value being 0.01 and word-match size between the query and database sequences being 7. The standard of potential mature miRNA sequences is that the length should be no less than 16 nt and no more than 25 nt, and the number of mismatches should be no more than 2. Secondly, precursor sequences of approximate 220 nt were extracted (100 nt upstream and 100 nt downstream to the BLAST hits) and utilized for the hairpin structure verification, testifying the exact length of the precursor sequences. These mature sequences were then BLASTXed by a web service to eliminate the protein-coding sequences. The remained precursor sequences underwent hairpin structure prediction through a web server called mfold. Besides, those EST sequences tagged with “3-” should be converted into the complementary chains which are the real EST sequences. Merely those fulfilling the criteria below were designated as qualified miRNAs: (1) predicted mature miRNAs need to have no more than 2 nt mismatches as compared with the known miRNAs, (2) the precursor of miRNA sequence could fold into a marked stem-loop hairpin secondary structure, (3) the mature miRNA sequence should be located in either arm of the hairpin structure, (4) miRNAs ought to have less than 6 mismatches with the opposite miRNA* sequence in the other arm, (5) no loops or breaks emerge in miRNA* sequences, and (6) the minimal folding free energy (MFE) of predicted pre-miRNA secondary structures has to be lower than −20 kcal/mol, while the minimal folding free energy index (MEFI) of it usually must be over 0.8 [17]. Also, the AU content of pre-miRNA within 30% to 70% was considered since the unstable structures of pre-miRNAs are needed to produce mature single-stranded miRNAs [18]. As a control, human EST database was also used to explore human miRNA before *M. fascicularis* analysis.

### 2.3. Targets' Analysis of Predicted miRNAs

Owing to the imperfect complementarity of animal miRNAs with their targets, it is difficult to judge the accuracy of the prediction. Complexity of regulation by miRNA-mediated targets at protein and mRNAs levels has made it more challenging to identify the targets [19]. We used the conserved binding seed-region, in which case those novel miRNAs probably from the same family could share the same seed region, to obtain the possible targeted genes with a web server of the widely used TargetScan. Then we input the list of targeted genes into another web server (Panther) which is designed for gene function cluster, and we could gain the protein class from the panther analysis result. After that, we clustered the same function class of protein with top 10 classes.

### 2.4. Phylogenetic Analysis of the New miRNAs

Considering the conservation of mature miRNAs among various species [20], both sequences of the novel and the known miRNAs in the same family were aligned and phylogenetically analyzed by MEGA 4 software to investigate their evolutionary relationships.

## 3. Results and Discussion

### 3.1. Prediction of * M. fascicularis* miRNAs

**Figure 2:**
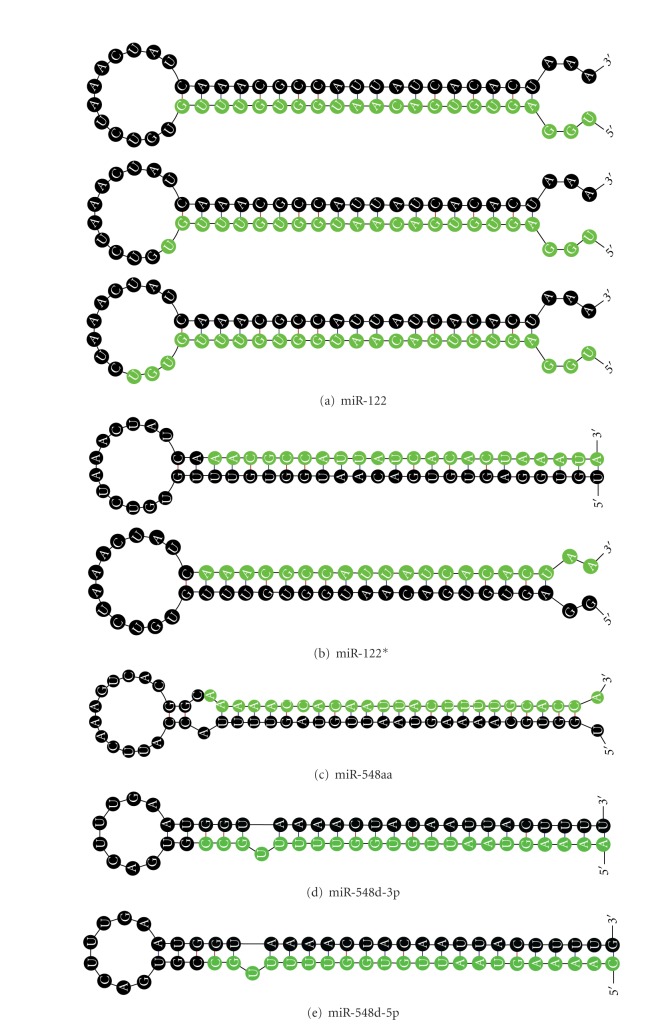

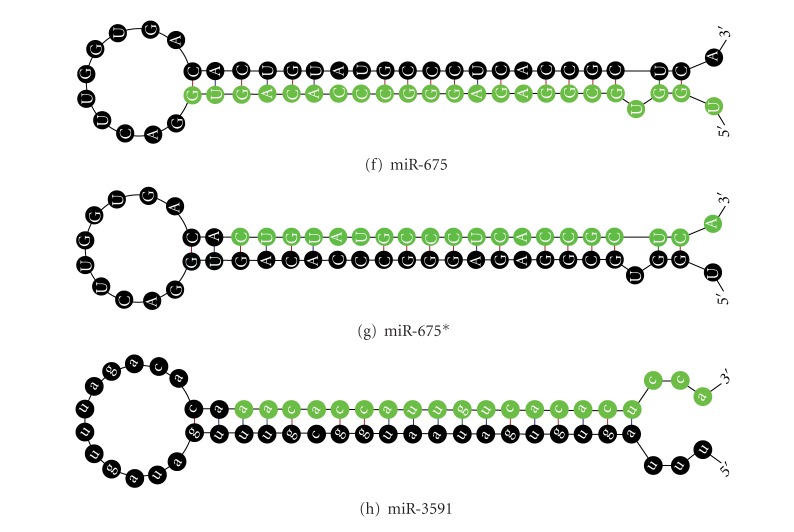
Secondary structures of *M. fascicularis* new miRNA precursors.

Sequence and structure verifications are the main theory based on the computer-based approach for miRNAs prediction. As described in the Materials and Methods section, after BLASTN searches, all blasted hits without protein-coding sequences were remained for secondary structure analysis; only those which satisfied with the filtering criteria were chosen as qualified candidates (Figure 1). We have firstly found the related experimentally annotated miRNAs from human EST data as control which can be seen in the Supplement 1 (see Supplementary Material available online at doi:10.1155/2012/957607), indicating this pipeline is valid for exploring candidate miRNAs. With this method, 8 potential *M*. *fascicularis *miRNAs were identified. Information on predicted *M*. *fascicularis *miRNAs, including names, lengths, original locations in the gene, sequences, and other aspects, was listed in Table 1, which is in agreement with the results predicted by other approaches [21–23] (data not shown). The length of the 8 predicted miRNAs ranged from 20 nt to 25 nt, while the precursor sequences ranged from 55 nt to 69 nt in length, all of which could shape into representative stem-loop structures, with the mature miRNA either on the 5′end or the 3′end (Figure 2). All the MFEIs of these hairpin structures were over 0.8, which was believed to be the essential standard to distinguish miRNAs from other RNAs [17]. Moreover, we mapped the *M. fascicularis *pre-miRNAs to the EST sequences and SRA sequences and we also found the location of EST sequences, in chromosome, which is listed in Table 2. For the close relationship between *M. fascicularis* and *Macaca mulatta* (*M. mulatta)*, we discovered that 95.90% of the miRNAs in *M. mulatta* share homologs in human miRNA, while 30.66% of the human miRNAs share homologs in *M. mulatta*, which situation may be seen very similarly in *M. Fascicularis* (Supplement 2). The *M. fascicularis* genome and next-generation sequencing are required for identifying other miRNAs not involving in its EST.

With the access to various sequence resources, bioinformatic approach-based miRNA identification is increasingly becoming superior to experimental methods owing to its advantages of low cost and high efficiency [24]. The number and sorts of miRNAs predicted in this work showed that this computer-based approach was as suitable and effective as that in this kind of work [25].

### 3.2. Prediction of *M. fascicularis* miRNA Targets

**Figure 3:**
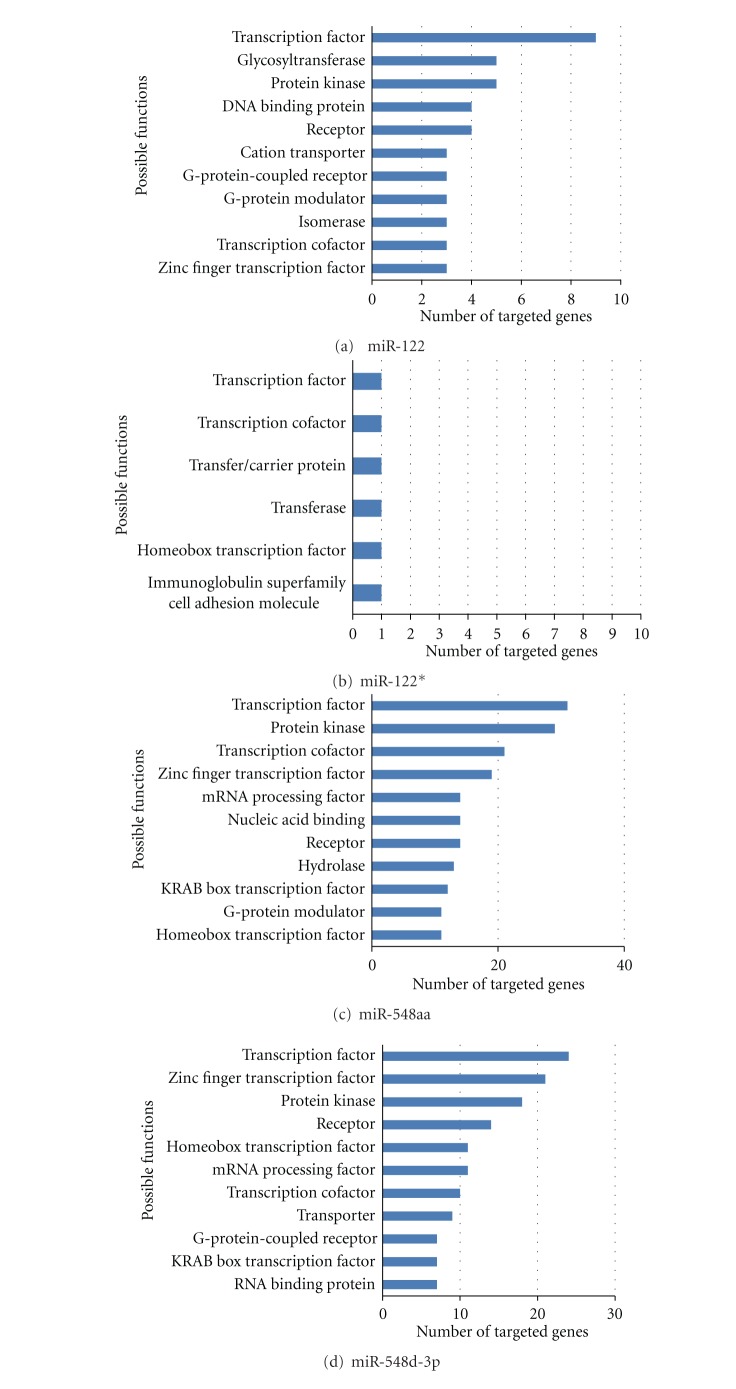

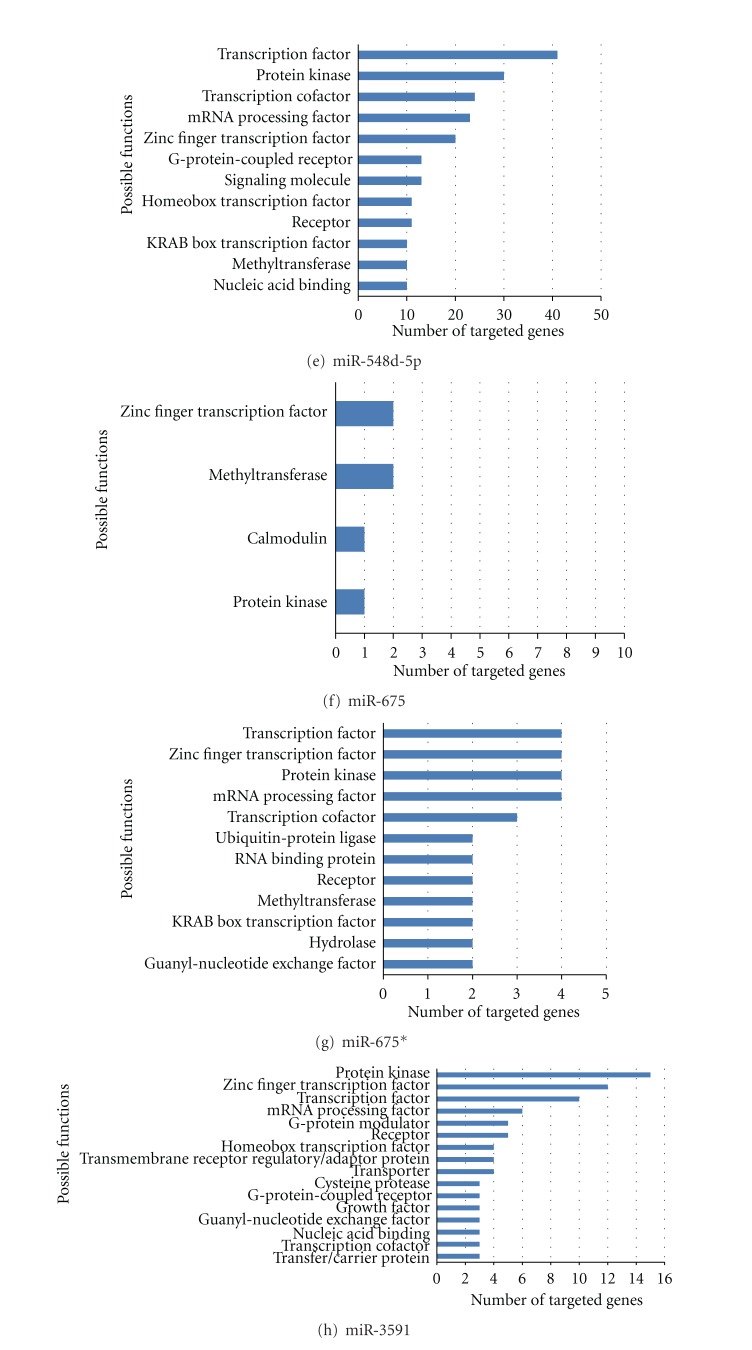
The major class of potential targets of newly identified miRNAs in *M. fascicularis. *

A total of 1,708 potential targeted proteins mainly for 8 *M. fascicularis *miRNAs were identified and the total targeted proteins were shown in Supplement 3. The major classes of potential targets of newly identified miRNAs in *M. fascicularis* are shown in Figure 3. These potential miRNA targets belong to a great many of gene families which play various roles during physiological and pathological processes.

Most of these targeted genes could express the transcription factors which controlled the expression of nearly all genes of *M. fascicularis.* Besides, another crucial part of the predicted targets as diverse sorts of enzymes such as protein kinases, which are particularly prominent in signal transduction and are known to regulate the majority of cell processes, such as cell growth, differentiation, metabolism, gene transcription. Protein kinases modify their target proteins by transferring phosphate groups from ATP to serine, threonine, or tyrosine residues on them (phosphorylation), which are one of the largest and most influential of gene families: constituting some 2% of the proteome, they regulate almost all biochemical pathways and may phosphorylate up to 30% of the proteome [26]. For the significant role that protein kinase plays in the physiological process, it suggests that miRNAs serve to orchestrate the activity of almost all cellular processes. Most of these novel miRNAs have shown important relationships with particular diseases or even certain physiological processes. MiR-122 is found to be enriched in liver cells where it has been implicated as a regulator of fatty acid metabolism in mouse studies [27]. MiR-548 genes are primate-specific and have many potential paralogs in the human genome. There are more than 3,500 putative miR-548 target genes; analysis of their expression profiles and functional affinities suggests cancer-related regulatory roles for hsa-mir-548 [28]. MiR-675 was found to be upregulated in human colon cancer cell lines and primary human colorectal cancer (CRC) tissues compared with adjacent noncancerous tissues [29].

It is generally accepted that the identification of genes targeted by the miRNAs above plays an essential role in figuring out the numerous functions of miRNAs in gene regulatory network. A bioinformatic method is definitely a vital step in discovering these targets, which is based on the homology between miRNAs and its potential targets. The application of protein databases from NCBI helped to predict the targeted genes in this work. With the increasingly wide use of web-based tool Targetscan, it offers an efficient way to acquire the functions of the targeted genes.

It also indicated that a single miRNA might regulate several target genes and vice versa. This result was compatible with other current studies in other species. Above all, it revealed that the research of miRNAs targeting ought to pay more attention on the whole system rather than on the one-to-one pattern between miRNAs and strongly predicted targets.

### 3.3. Phylogenetic Analysis of the Novel miRNAs

The result of phylogenetic analysis was demonstrated in Supplement 4. Becausewe only could find the sequences of hsa-miR-3591 and rno-miR-3591 in the miRBase database, with the predicted miR-3591, which could hardly be constructed into a phylogenetic tree. For the same reason, we only could get the sequences of has-miR-548d-1, has-miR-548d-2, mml-miR-548d, and has-miR41-548d-3p; at last we decided to put them in a phylogenetic tree of miR-548d family. So we gained 5 phylogenetic trees of mature miRNA sequences and 3 phylogenetic trees of precursor miRNA sequences.

It has been proven that mature miRNAs as well as precursors are both highly conserved among various species of the same kingdom. Because miRNAs are evolutionarily conserved regulators of gene expression, it is expected that their pre-miRNAs should have respective phylogenetic orthologs [21]. One research group has found that comparison of the precursor sequences of the predicted *Vigna unguiculata* miRNAs with other members in the same family showed that most members could be found to have a high degree of sequence similarity with others. Also, miRNAs might evolve at different rates not only within the same plant species but also in different ones [30]. Another group discovered that miRNAs in the same family from different species are highly conserved and share the same DNA sequence. So miRNAs may have the same ancestor in very early evolution [31]. Sequence comparisons of the members in the same miRNA families showed that all of the mature miRNAs of *M. fascicularis* had a high sequence similarity with the other members. Besides, the phylogenetic analysis of precursors could show the different locations of the miRNAs which are from the same family because compared with mature miRNA the other parts of the precursor sequence are less conserved, which is consistent with the report of Wang et al. [32]. Previous results have demonstrated that there is a direct correlation between the number of miRNAs and morphological complexity [33]. Based on the close relationship between *M. fascicularis* and human shown in phylogenic trees, many miRNAs are supposed to be involved in *M. fascicularis*. Other miRNAs not located in *M. fascicularis* EST database are able to be determined by other approaches such as Solexa deep sequencing when its genome is available. Together, the discovery of miRNAs has provided another kind of genetic material for the evolution investigation of *M. fascicularis*.

## 4. Conclusions

In the present study, with a new published bioinformatic method, a total number of 8 potential miRNAs were identified by the analysis of EST databases of *M. fascicularis*. Most of these targeted genes are related to transcription factors and protein kinases. The findings from this study are believed to contribute to further researches on the function and regulatory mechanisms of miRNAs in *M. fascicularis*. 

## Supplementary Material

Percentage of human miRNAs represented in the *Macaca mulatta* miRNAs database, and vice versa.
Phylogenetic analysis of maturemiRNAs sequences in different families. (A) miR-122(B) miR-122*(C) miR-548aa (D) miR-548d (E) miR-675
Phylogenetic analysis of precursor miRNAs sequences in different families. (A) pre-miR-122 family (B) pre-miR-548 family (C)pre-miR-675 family

## Figures and Tables

**Table 1:** Novel miRNAs identified in *Macaca* *fascicularis*.

miRNA	Homolog miRNA	EST name	miRNA sequence (5^′^-3^′^)	Position	NM/nt	LM/nt	LP/nt	A + U(%)	MFE	MFEI
miR-122	hsa	BB880656	UGGAGUGUGACAAUGGUGUUUG	176–155	0	22	56	62.50	−24.2	1.15
	gga	BB891562	UGGAGUGUGACAAUGGUGUUUGU	174–152	0	23	56	62.50	−24.2	1.15
	mdo	BB891562	UGGAGUGUGACAAUGGUGUUUGUGU	174–150	0	25	57	62.50	−24.2	1.13
miR-122^∗^	hsa	BB891562	AACGCCAUUAUCACACUAAAUA	138–117	0	22	60	63.33	−25.3	1.10
	mmu	BB891562	AAACGCCAUUAUCACACUAA	139–120	0	20	54	61.11	−24.2	1.15
miR-548aa	hsa	BB883545	AAAAACCACAAUUACUUUUGCACCA	592–568	0	25	67	64.18	−29.9	1.20
miR-548d-5p	hsa	BB883545	AAAAGUAAUUGUGGUUUUUGCC	574–595	1	22	55	70.91	−25.1	1.57
miR-548d-3p	mml	BB883545	CAAAAACCACAAUUACUUUUGC	593–572	1	22	57	68.42	−27.2	1.51
miR-675	hsa	DC648667	UGGUGCGGAGAGGGCCCACAGUG	562–584	0	23	55	38.18	−29.2	0.86
miR-675^∗^	hsa	DC648667	CUGUAUGCCCUCACCGCUCA	597–616	0	20	55	38.18	−29.2	0.86
miR-3591	rno	BB891562	AACACCAUUGUCACACUCCA	155–174	0	20	57	62.50	−23.6	1.12

NM: nucleotide mismatch; LM: length of mature miRNA; LP: length of miRNA precursor; MFE: minimum free energy; MFEI: minimum free energy index.

**Table 2:** Chromosome location of pre-miRNAs.

Pre-miRNA	EST name	Position	Chromosome location of EST	SRA sequence	Position
Pre-miR-122	BB880656	121–176	18: 5318–6012	SRR223515.5362734	41–96
	BB891562	119–174	18: 5224–5998	SRR223515.7331439	125–180
Pre-miR-122^∗^	BB891562	117–176	18: 5224–5998	SRR223515.7331439	123–182
	BB891562	120–173	18: 5224–5998	SRR223515.7331439	126–179
Pre-miR-548aa	BB883545	568–634	16: 2735–2669	—	—
Pre-miR-548d-5p	BB883545	574–628	16: 2656–3048	—	—
Pre-miR-548d-3p	BB883545	283–339	16: 2656–3048	—	—
Pre-miR-675	DC648667	520–584	—	SRR223514.4470015	3–67
Pre-miR-675^∗^	DC648667	562–616	—	SRR223513.10298212	133–187
Pre-miR-3591	BB891562	119–175	18: 5224–5998	SRR223515.7331439	125–181

^∗^All matched SRA sequences are shown in Supplement 5; —not found.
