# Supplementary material for: Identification and Characterization of MicroRNAs in Macaca fascicularis by EST Analysis
Source: Comp Funct Genomics. 2012 Jul 5;2012:957607. doi: 10.1155/2012/957607 (PMC3398586; doi:10.1155/2012/957607)

Supplementary Data:

Table. Percentage of human miRNAs represented in the *Macaca mulatta* miRNAs database, and vice versa.

Species	Number of homologs	Percent(Homologs/Total miRNAs)
mml-miRNA	468	95.90%(468/488)
hsa-miRNA	589	30.66%(589/1921)

Phylogenetic analysis of maturemiRNAs sequences in different families. (A) miR-122(B) miR-122*(C) miR-548aa (D) miR-548d (E) miR-675

(A) miR-122

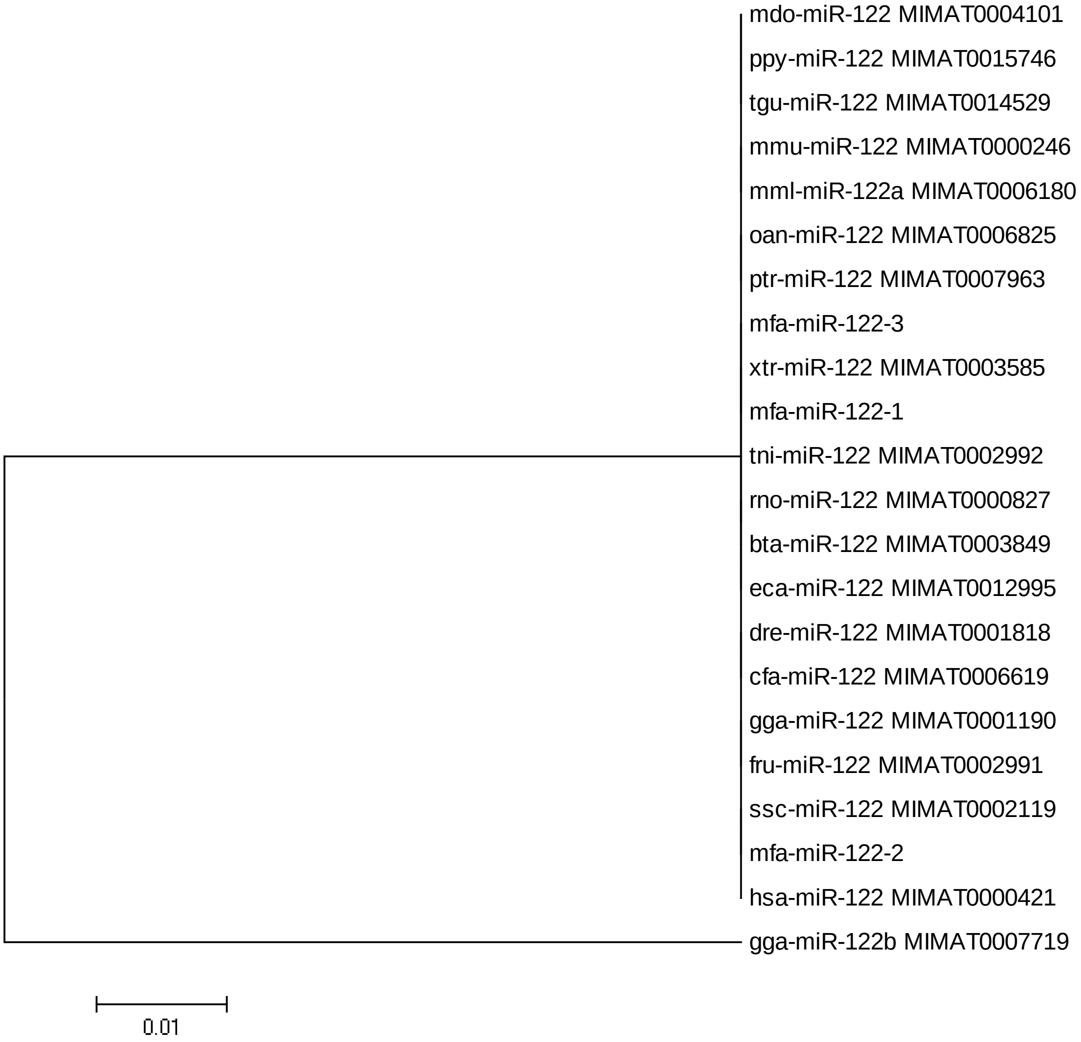
mdo-miR-122 MIMAT0004101
ppy-miR-122 MIMAT0015746
tgu-miR-122 MIMAT0014529
mmu-miR-122 MIMAT0000246
mml-miR-122a MIMAT0006180
oan-miR-122 MIMAT0006825
ptr-miR-122 MIMAT0007963
mfa-miR-122-3
xtr-miR-122 MIMAT0003585
mfa-miR-122-1
tni-miR-122 MIMAT0002992
rno-miR-122 MIMAT0000827
bta-miR-122 MIMAT0003849
eca-miR-122 MIMAT0012995
dre-miR-122 MIMAT0001818
cfa-miR-122 MIMAT0006619
gga-miR-122 MIMAT0001190
fru-miR-122 MIMAT0002991
ssc-miR-122 MIMAT0002119
mfa-miR-122-2
hsa-miR-122 MIMAT0000421
gga-miR-122b MIMAT0007719
0.01

(B) miR-122*

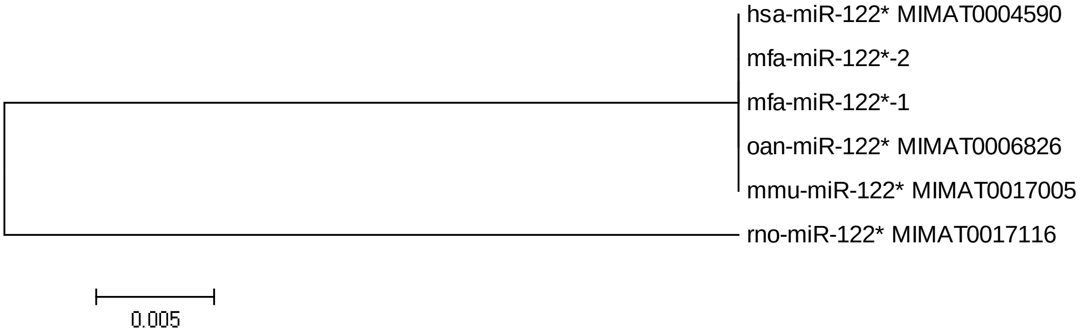
hsa-miR-122* MIMAT0004590
mfa-miR-122*-2
mfa-miR-122*-1
oan-miR-122* MIMAT0006826
mmu-miR-122* MIMAT0017005
rno-miR-122* MIMAT0017116
0.005

(C) miR-548aa

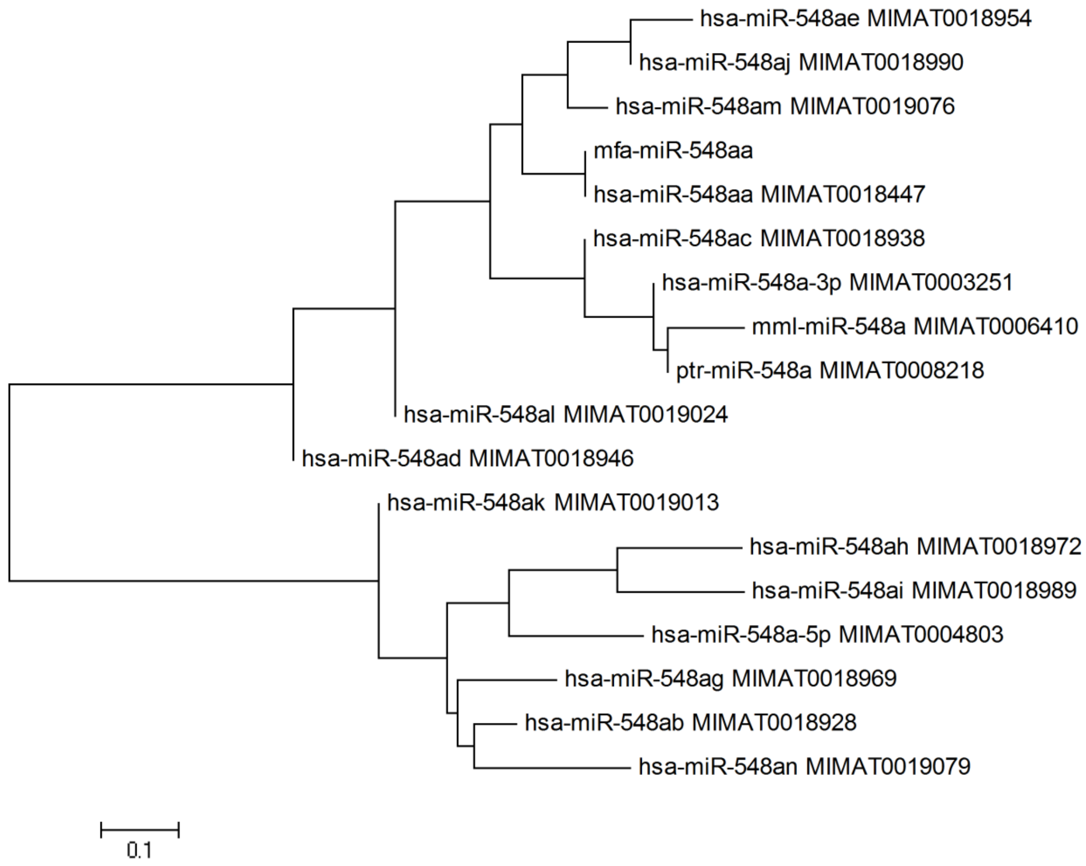

(D) miR-548d

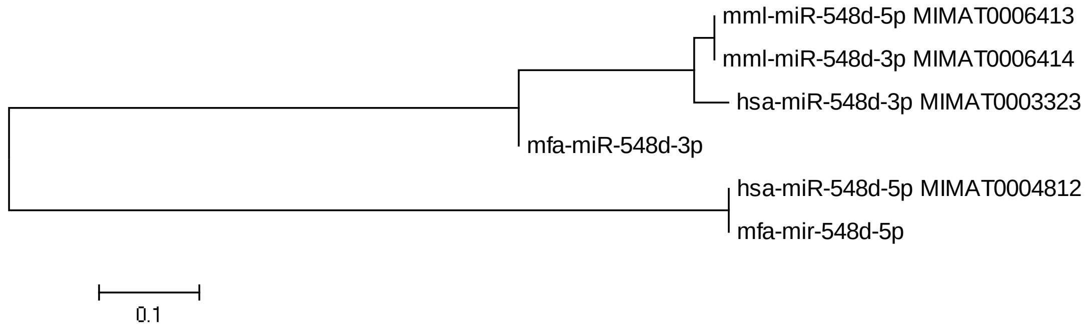

(E) miR-675

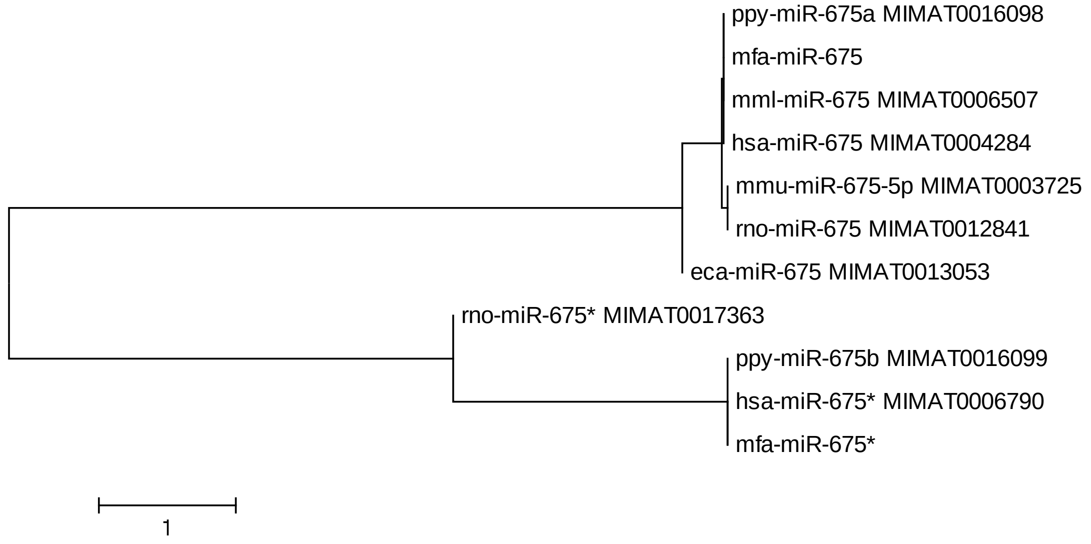
ppy-miR-675a MIMAT0016098
mfa-miR-675
mml-miR-675 MIMAT0006507
hsa-miR-675 MIMAT0004284
mmu-miR-675-5p MIMAT0003725
rno-miR-675 MIMAT0012841
eca-miR-675 MIMAT0013053
rno-miR-675* MIMAT0017363
ppy-miR-675b MIMAT0016099
hsa-miR-675* MIMAT0006790
mfa-miR-675*
1

Phylogenetic analysis of precursor miRNAs sequences in different families. (A) pre-miR-122 family (B) pre-miR-548 family (C)pre-miR-675 family

(A) pre-miR-122 family

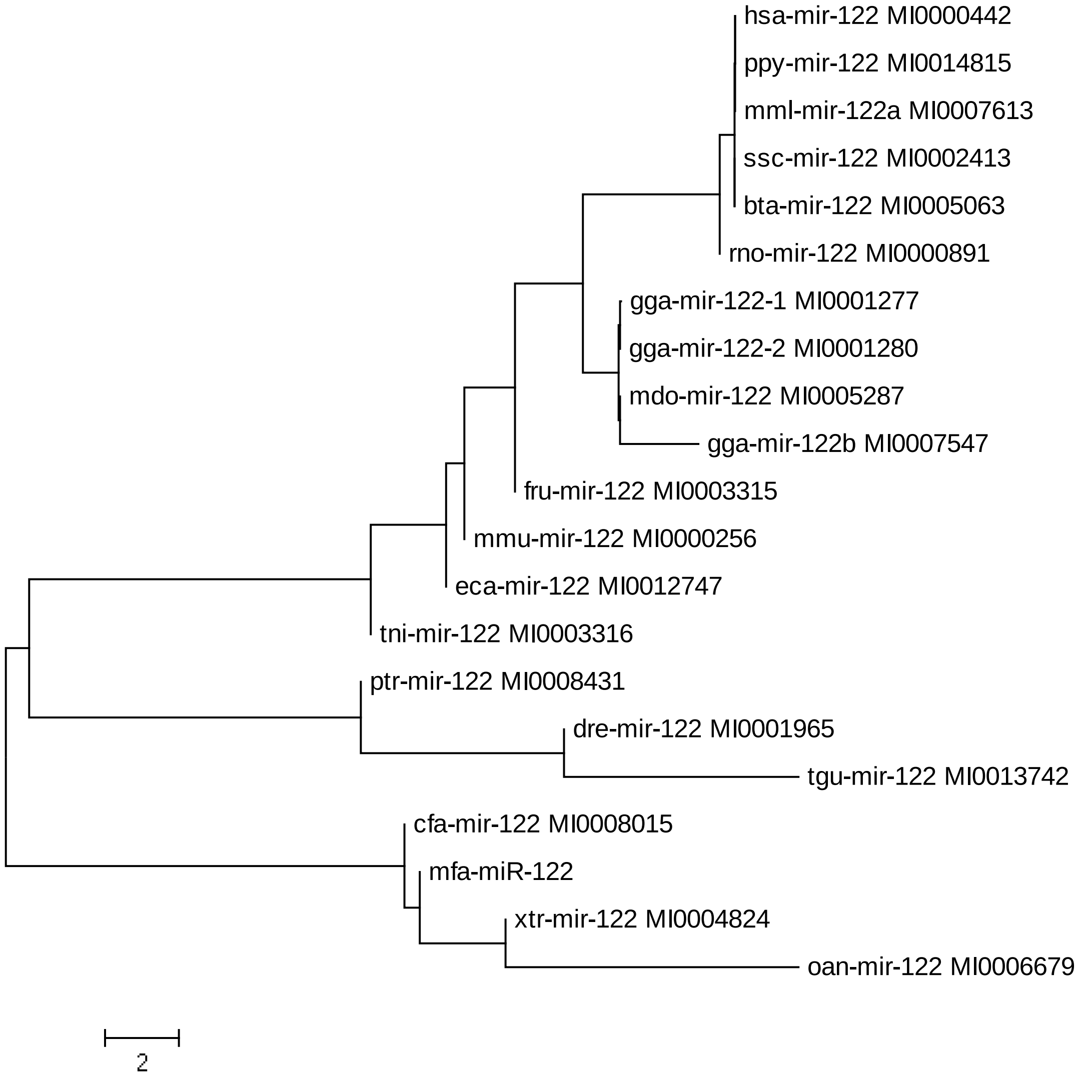

(B) pre-miR-548 family

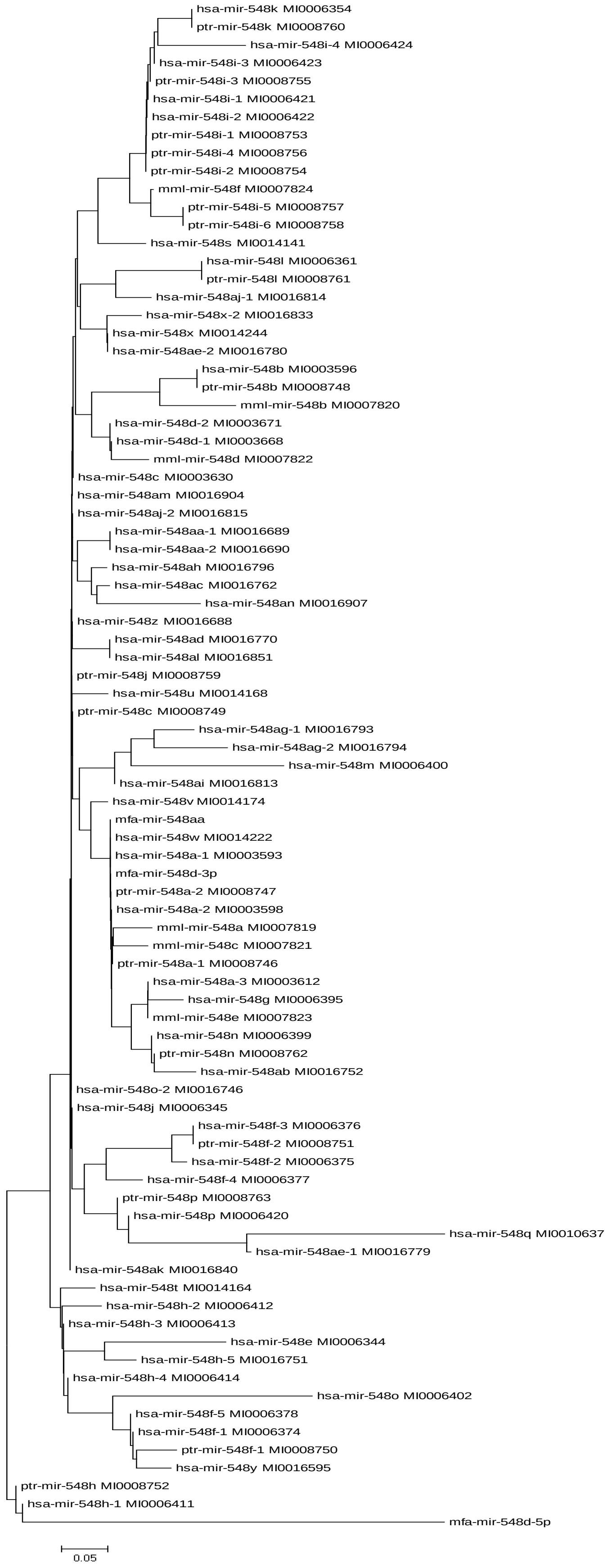

(C) pre-miR-675 family

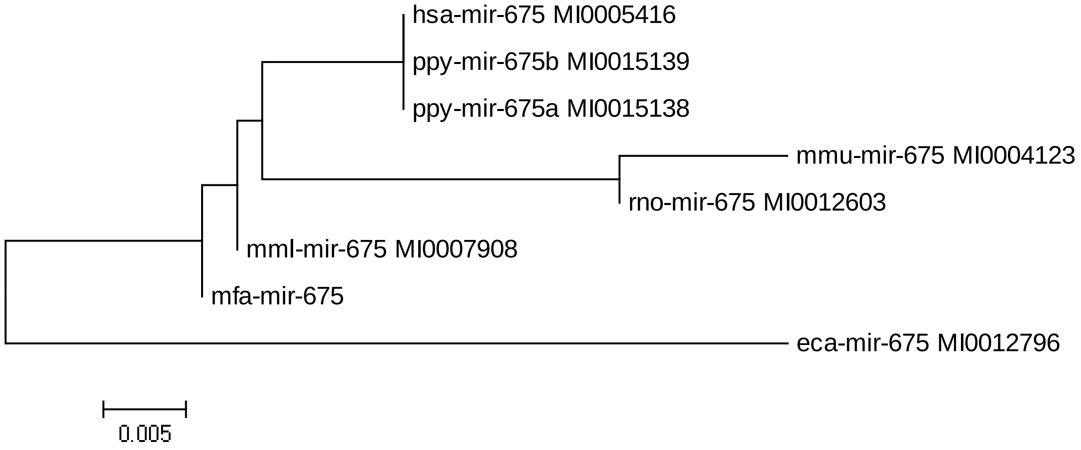

Supplement: Supplementary file 1 — Percentage of human miRNAs represented in the Macaca mulatta miRNAs database, and vice versa. Phylogenetic analysis of maturemiRNAs sequences in different families. (A) miR-122(B) miR-122*(C) miR-548aa (D) miR-548d (E) miR-675 Phylogenetic analysis of precursor miRNAs sequences in different families. (A) pre-miR-122 family (B) pre-miR-548 family (C)pre-miR-675 family [file 957607.f1.pdf]
